# Non-contrast coronary magnetic resonance angiography: current frontiers and future horizons

**DOI:** 10.1007/s10334-020-00834-8

**Published:** 2020-04-02

**Authors:** Yoko Kato, Bharath Ambale-Venkatesh, Yoshimori Kassai, Larry Kasuboski, Joanne Schuijf, Karan Kapoor, Shelton Caruthers, Joao A. C. Lima

**Affiliations:** 1 Division of Cardiology, Johns Hopkins University School of Medicine, 600 N Wolfe St, Blalock 524, Baltimore, MD 21287-0409 USA; 2 Division of Radiology, Johns Hopkins University, Baltimore, MD USA; 3 Canon Medical Systems Corporation, Otawara, Japan; 4 Canon Medical Research USA, Inc., Cleveland, OH USA

**Keywords:** Magnetic resonance coronary angiography, Image acceleration technique, Image denoising, Review article

## Abstract

Coronary magnetic resonance angiography (coronary MRA) is advantageous in its ability to assess coronary artery morphology and function without ionizing radiation or contrast media. However, technical limitations including reduced spatial resolution, long acquisition times, and low signal-to-noise ratios prevent it from clinical routine utilization. Nonetheless, each of these limitations can be specifically addressed by a combination of novel technologies including super-resolution imaging, compressed sensing, and deep-learning reconstruction. In this paper, we first review the current clinical use and motivations for non-contrast coronary MRA, discuss currently available coronary MRA techniques, and highlight current technical developments that hold unique potential to optimize coronary MRA image acquisition and post-processing. In the final section, we examine the various research-based coronary MRA methods and metrics that can be leveraged to assess coronary stenosis severity, physiological function, and atherosclerotic plaque characterization. We specifically discuss how such technologies may contribute to the clinical translation of coronary MRA into a robust modality for routine clinical use.

## Background

The diagnosis and management of coronary artery disease (CAD) and consequent myocardial ischemia are central to the prevention of future cardiac events. In this regard, the main advantage of non-contrast coronary magnetic resonance angiography (coronary MRA) is its ability to assess coronary artery morphology and function without ionizing radiation or contrast media. Despite the early recognition of such extraordinary potential [1], significant technical limitations including reduced spatial resolution, long acquisition time, and low signal-to-noise ratio impairing image quality have caused coronary MRA to be less preferred than competing non-invasive techniques such as coronary computed tomography angiography (CTA), and prevented it from routine clinical utilization [2, 3]. However, despite these technical limitations, coronary MRA has contributed significantly to our current understanding of CAD pathophysiology [4, 5] by providing insights into coronary artery distensibility in response to stress [6], plaque characteristics [7], and plaque inflammation [8]. Its usefulness as a non-invasive research method to assess CAD in different groups of patients has been demonstrated not only in single center clinical investigations [9, 10], but also in multi-center studies [11, 12]. Moreover, coronary MRA has proven to be important in the delineation of congenital coronary abnormalities, for which it is recommended as the clinical modality of choice, particularly when there is concern about the use of radiation and contrast [13, 14]. Recent developments in magnetic resonance imaging are poised to specifically impact coronary MRA in its ability to assess coronary anatomy and function in patients with chest pain or other clinical manifestations that suggest the presence of CAD [15–18]. More recently, the possibility of using deep learning techniques to enhance image quality in applications characterized by low signal-to-noise ratios has opened additional avenues of potential development in coronary MRA imaging [19, 20]. In this paper, we will first review the current clinical status of non-contrast coronary MRA, and then discuss current technical efforts to optimize coronary MRA image acquisition and post-processing. In the last section of this paper, we will discuss the various techniques of coronary anatomical and functional assessment on MRI that when combined together promise diagnostic and prognostic performance boost. Through these discussions, we hope to guide the readers to realize the promise of coronary MRA as a diagnostic tool for clinical use.

## Current status of non-contrast coronary MRA

### Motivations for the clinical use of non-contrast coronary MRA

CAD remains the leading cause of death in the world [21]. Catheter-based X-ray coronary angiography (CAG) is the current gold standard for the diagnosis of significant (> 50% diameter stenosis) CAD. However, around half of the patients referred for diagnostic CAG do not have significant stenosis [22–24], yet are exposed to ionizing radiation and contrast media as well as the potential risks associated with this invasive procedure [25]. The discomfort of patients during the invasive procedure is also not negligible. Non-contrast coronary MRA is an attractive option for anatomical coronary artery assessment in this regard, albeit relatively underdeveloped compared to coronary CTA. However, there are advantages non-contrast coronary MRA holds over CTA that may be leveraged as the technique matures including: (1) “one-stop-shop-test” by combining it with additional anatomical and functional MRI methods, (2) robustness to the calcium “blooming” that hampers CTA assessment, and (3) absence of ionizing radiation or contrast media exposure [9].

**Fig. 1:**
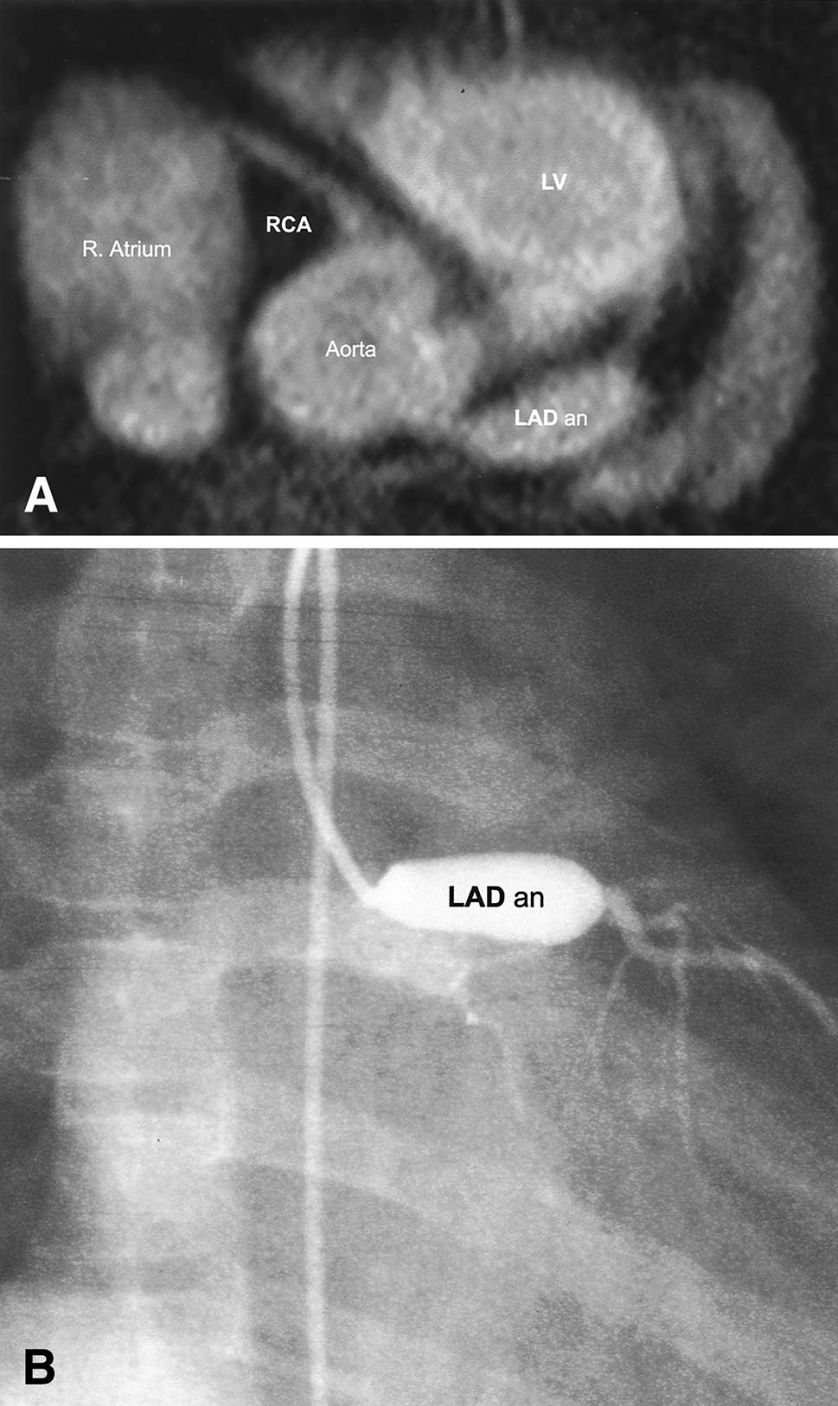
Coronary MRA (**a**) and CAG (**b**) image of a left anterior descending coronary artery aneurysm (LAD an) in a patient with Kawasaki disease. *MRA* magnetic resonance angiography, *CAG* coronary angiography, *LAD* left anterior descending, *LV*  left ventricle, *R. Atrium* right atrium, *RCA* right coronary artery. (Reprinted with permission from Mavrogeni et al. [30], Copyright © 2004 by the American College of Cardiology Foundation)

There are several well-defined patient populations that benefit from these advantages. Pediatric congenital heart disease patients frequently present for evaluation of coronary anatomy post-surgery or suspected coronary anomaly. These pediatric patients require multiple follow-up examinations and thus are good candidates for non-contrast coronary MRA [26]. Albrecht et al. have reported that in a pediatric population with suspected anomalous coronary arteries, coronary MRA provided comparable diagnostic accuracy with coronary CTA in the detection of findings that occurs in proximal to mid main coronary arteries like anomalies, high origin, and inter-arterial course of the coronary arteries, admitting superior visualization with CTA in distal coronary arteries [27]. Meanwhile, drawbacks for coronary MRA in pediatric patients include smaller coronary diameter, high resting heart rate, and difficulty of keeping the same position for a long time as compared to adults. Cardiac MRI with sedation or coronary CTA with its inherently higher spatial resolution may be considered appropriate in some cases [28, 29]. Evaluation of Kawasaki disease is another accepted indication for which coronary MRA is reported to be equivalent to CAG (Fig. 1) [30, 31]. Indeed, coronary MRA is recommended as the clinical modality of choice for these populations in which repeated radiation and contrast exposure are major concerns [13, 14]. The Japanese Circulation Society guidelines for Kawasaki disease in 2013 stipulate that coronary MRA is preferred over coronary CTA, as it allows repeated imaging while heart rate control is not required, which enables infants and young children to undergo the examination during sleep [32]. On the other hand, while the European Society of Cardiology guidelines for adult congenital disease published in 2010 recommend regular usage of cardiovascular magnetic resonance when considered superior to echocardiography, these guidelines do admit that CT is superior for non-invasive coronary angiography [33].

**Fig. 2:**
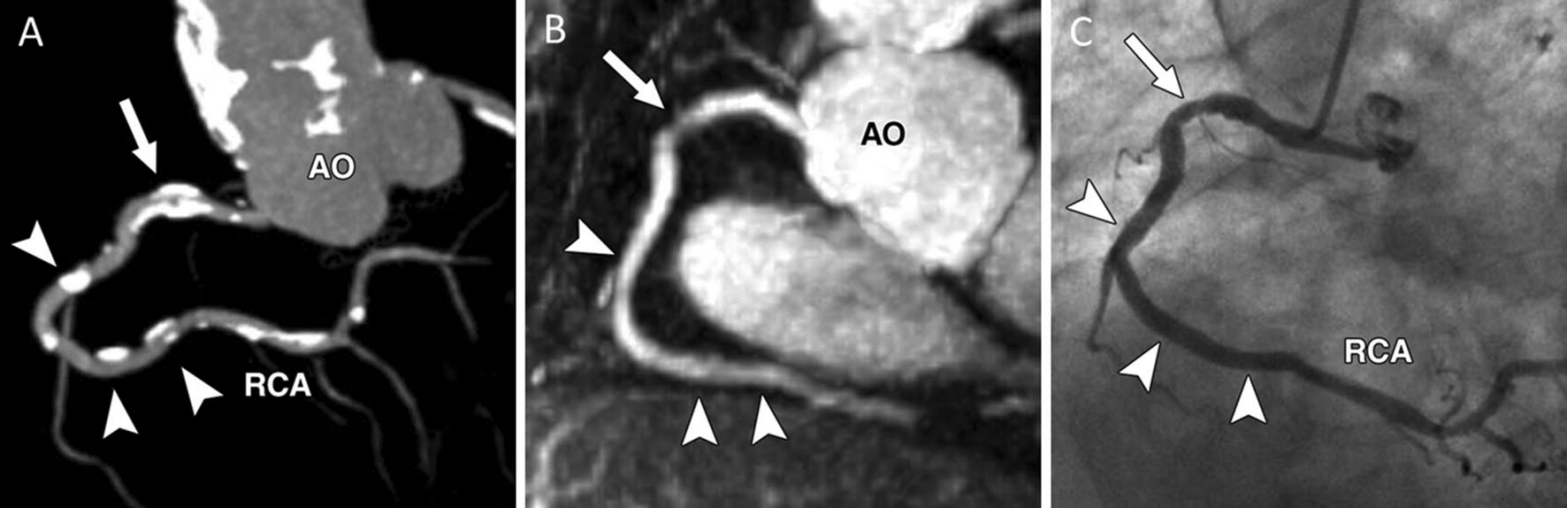
Representative images of the RCA in three different modalities of **a** coronary CTA, **b** coronary MRA, and **c** CAG. **a** Diffuse calcification (arrow and arrowheads) was detected in RCA on coronary CTA MIP image. Coronary MRA MIP image **b** shows moderate stenosis (arrow, **b**) and CAG (**c**) confirms moderate stenosis (arrow, **c**) in corresponding segment where heavy diffuse calcification can be seen in **a** (arrow in **a**). **b**, **c** No significant stenosis (arrowheads) in corresponding segments where nodal calcifications are located in (**a**). *AO* aorta, *RCA* right coronary artery, *CTA* computer tomography angiography, *MRA* magnetic resonance angiography, *CAG* coronary angiography, *MIP* maximum intensity projection. (Reprinted with permission from Liu et al. [37], Copyright © 2007 by the American Roentgen Ray Society, ARRS)

In chronic kidney disease (CKD) patients, contrast administration is a major concern due to the risk of post-contrast acute kidney injury after iodine-based contrast media injection [34, 35] or nephrogenic systemic fibrosis after gadolinium-based contrast media [36]. Moreover, these patients often require multiple follow-ups since they frequently present with severely calcified plaques in the coronary arteries. Not only does non-contrast MRCA take away the risk of further kidney injury by contrast injection, but it also allows for coronary lumen visualization without blooming artifacts from calcium as seen in coronary CTA. In MRI, calcifications present very low signal both on T1 and T2 images due to their low proton density. As a result, coronary calcifications do not obscure the coronary lumen in MRI. Indeed, coronary MRA has been shown to have a better performance in the detection of significant stenosis in patients with moderate to severe calcifications than CTA (Fig. 2) [37].

The principal idea for the treatment strategy of stable CAD is 1) invasive revascularization for the left main stenosis and 2) invasive revascularization when symptoms such as chest pain remain despite optimal medical therapy. Coronary MRA provides information on the distribution and severity of stenotic lesions, which is helpful to assess left main lesions, or when deciding the treatment strategy between percutaneous catheter intervention (PCI) and coronary artery bypass graft (CABG) surgery.

Another recent concern is the possibility of gadolinium depositions in the brain. This phenomenon has been correlated with the number of previous examinations involving gadolinium-based contrast administration [38] and has also been reported in subjects without severe renal dysfunction [39]. Although the long-term effects are not clear, the general consensus is that when possible, reduced exposure to gadolinium is preferable. Non-contrast coronary MRA is in line with this principle.

Meanwhile, coronary MRA has a few major limitations including (1) time-consuming image acquisition which takes around 10–20 min, (2) low spatial resolution (around 1-2 mm) compared to coronary CTA (around 0.5 mm) or CAG(< 0.3 mm), (3) poor visualization of coronary stents or calcified plaque due to the low proton density of these elements and limited visibility of the stent lumen due to radiofrequency (RF) shielding effects [40], susceptibility artifacts by diamagnetic (calcium) or ferromagnetic (stent) effect (though manageable with short echo time setting [41]), and (4) no consensus on coronary MRA post-processing and analysis methodology.

As these challenges are tackled and the technique matures, integrated protocols in which coronary MRA is added to other cardiac MRI examinations as a “one-stop-shop-test” are likely to improve the diagnostic and prognostic performance of the MRI examination. For example, the addition of free-breathing whole heart contrast-enhanced coronary MRA at 3 T to the combination of stress/rest myocardial perfusion imaging and late gadolinium enhancement (LGE) image significantly improved the sensitivity and diagnostic accuracy of detection of ≥ 50% stenosis as diagnosed by CAG (per-patient bases, sensitivity 100% vs. 76.5%, *p* < 0.01, accuracy 89.1% vs. 73.9%, *p* < 0.01) [42]. Although not specifically shown, a similar benefit of non-contrast coronary MRA is highly expected. Interestingly, the additive value of coronary MRA was not significant in another study that evaluated the integration of non-contrast coronary MRA with myocardial perfusion imaging (MPI) and LGE at 1.5 T for the detection of hemodynamically significant stenosis, defined as either a severe lesion of ≥ 90% luminal narrowing/occlusion or flow reserve ≤ 0.80 [43]. This result suggests that the diagnostic performance of MPI and LGE for the detection of physiological ischemia is already saturated and therefore there is less room for the contribution of the morphological assessment to significantly improve the diagnostic performance. The potential additive value of MRCA remains in excluding three-vessel disease in the differential diagnosis with microvascular disease, which was not assessed in this study.

Prognostication of cardiac events is a primary contribution of non-contrast coronary MRA by itself or in combination with other cardiac MRI phenotypes as a “one-stop-shop-test”. Yoon et al. have reported during a median follow-up of 25 months of 207 patients that the presence of significant stenosis detected on 1.5 T non-contrast coronary MRA was significantly associated with all cardiac events (hazard ratio = 20.78, *p* = 0.001) [44].

### Preparation for the clinical coronary MRA acquisition

The current acquisition standard for coronary MRA—three-dimensional (3D) free-breathing whole-heart coverage coronary MRA, requires several preparatory techniques such as electrocardiography (ECG) and respiratory gating [45]. A patient-specific acquisition window is set based on ECG gating during either the diastolic or systolic phase, corresponding to the phase with the least coronary artery motion. For the static phase selection, transaxial cine MR images with a steady-state free precession (SSFP) sequence are acquired prior to the coronary MRA acquisition to evaluate the motion pattern of the right coronary artery (RCA). The dome of the right hemidiaphragm is the preferred location of the respiratory navigator, while the details of navigator implementation tend to be vendor specific [45]. Although the image gets sharper when the ECG and respiratory gating width are narrowed, the inherent disadvantages of narrow windows are a reduction in the data acquisition success rate and a corresponding increase in the image acquisition time, potentially leading to more disturbance from patient motion. In the respiratory navigator, the current general setting of a small gating window of 5–6 mm leads to a low imaging efficiency (30–50%) [46]. In the absence of overt contraindications, the administration of sublingual nitroglycerin (NTG) is recommended to improve luminal visualization in terms of signal-to-noise ratio (SNR), vessel diameter, and vessel sharpness of the coronary MRA [47]. Heer et al. have reported that the significant increase in coronary diameter and visible vessel length observed with sublingual NTG administration result in improved sensitivity, specificity, and diagnostic accuracy for the detection of > 50% coronary stenosis on 1.5 T non-contrast coronary MRA [48].

### 1.5 T vs. 3 T coronary MRA

Coronary MRA at higher magnetic field strengths has been an area of active research given the potential benefits in SNR and contrast-to-noise ratio (CNR) as well as higher spatial and temporal resolutions. The optimal non-contrast coronary MRA imaging technique differs between 1.5 T MRI and 3 T MRI. In 1.5 T-MRI, balanced steady-state free precession imaging (bSSFP) is the most commonly used sequence [12, 45, 49]. However, the applicability of bSSFP to 3 T MRI is limited for a variety of reasons including (1) more pronounced B0 and B1 field inhomogeneities than 1.5 T, (2) degraded image quality and increased magnetic field heterogeneity from RF pulse-induced dielectric effects, and (3) increased power deposition in the human body at 3 T limits the use of large flip angles for SSFP imaging [50]. To address the above-mentioned challenges, spoiled gradient echo sequences are used [51, 52]. Despite somewhat limited by lower SNR and CNR than SSFP, spoiled gradient echo sequencing, ECG- and diaphragm navigator gating, and fat suppression have become the standard acquisition protocol for non-contrast coronary MRA at 3 T [15]. In a study that investigated the image quality between SSFP and gradient echo sequence for coronary MRA at 3 T, the image quality was higher and the measured vessel length was longer in gradient echo sequence [53]. When performed with the same sequence, 3 T coronary MRA is not inferior to 1.5 T coronary MRA both in image quality and diagnostic accuracy for the detection of coronary stenosis [54].

**Fig. 3:**
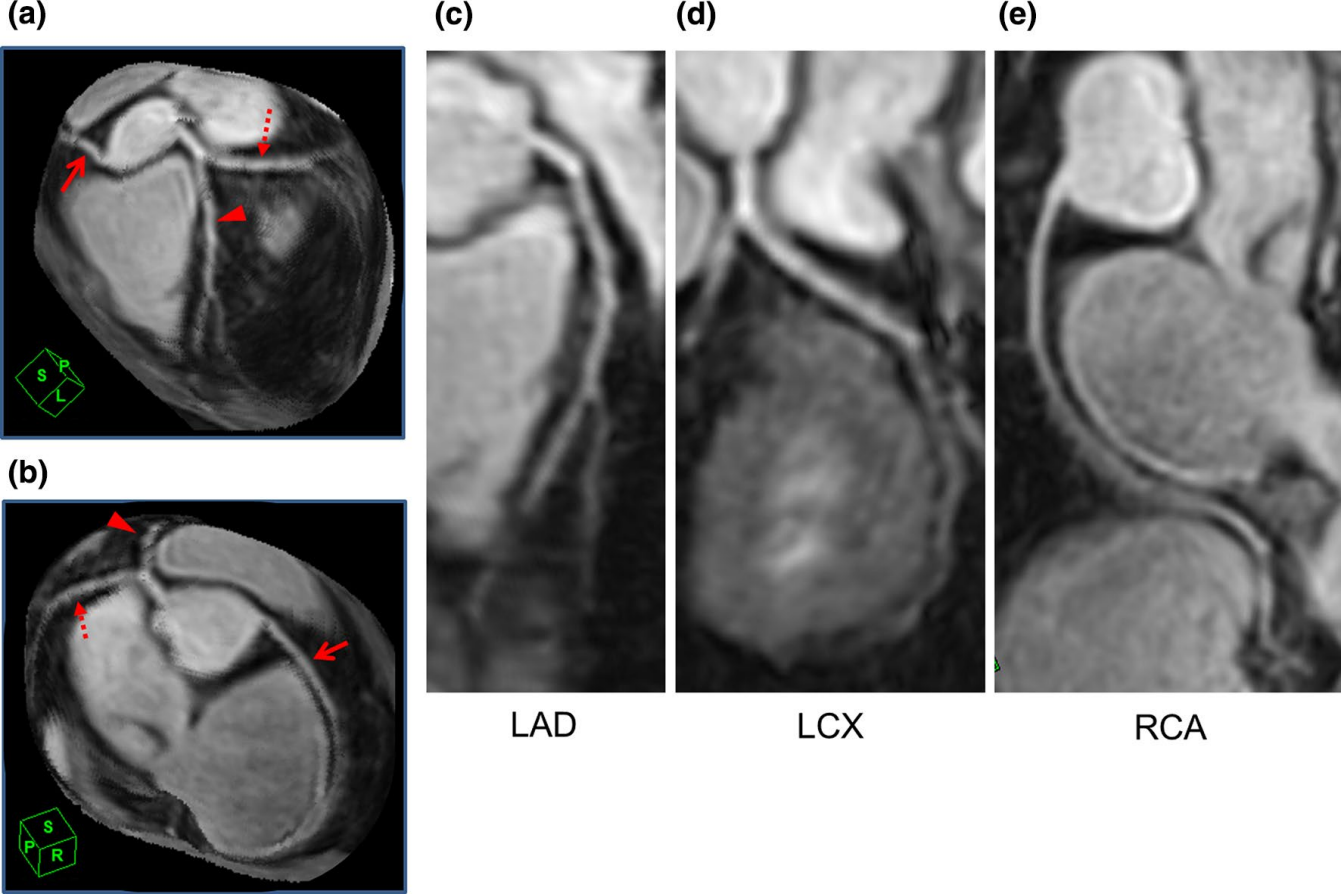
A representative case of a non-contrast coronary MRA with a conventional technique. A non-contrast coronary MRA acquired in 3 T scanner with the conventional image acquisition acceleration method of parallel imaging (PI) is presented. A spoiled gradient echo sequence with ECG gating, diaphragm navigator gating, and fat suppression with spectral attenuated inversion recovery was used for the image acquisition. The MRI acquisition parameters were FOV = 350 × 350 mm, matrix = 224 × 232, slice thickness = 1.5 mm, slice number = 80, acceleration factor = 2.0 × 2.0, TR = 4.9 ms, TE = 1.9 ms, flip angle = 12°, bandwidth = 326 Hz/pix, acquisition voxel size = 1.6 × 1.5 × 1.5 mm, reconstructed voxel size = 0.80 × 0.80 × 0.75, navigator gating window = 5 mm. **a**, **b** Volume rendering images. Arrowheads: LAD, dashed arrows: LCX, and solid line arrows: RCA. **c**–**e** Curved MPR images of **c** LAD, **d** LCX, and **e** RCA. *MRA* magnetic resonance angiography, *LAD* left anterior descending, *LCX* left circumflex, *RCA* right coronary artery

Figure 3 shows a representative non-contrast coronary MRA case acquired in 3 T MRI with the conventional image acquisition acceleration method of parallel imaging (PI).

### Non-contrast vs. contrast-enhanced coronary MRA

**Fig. 4:**
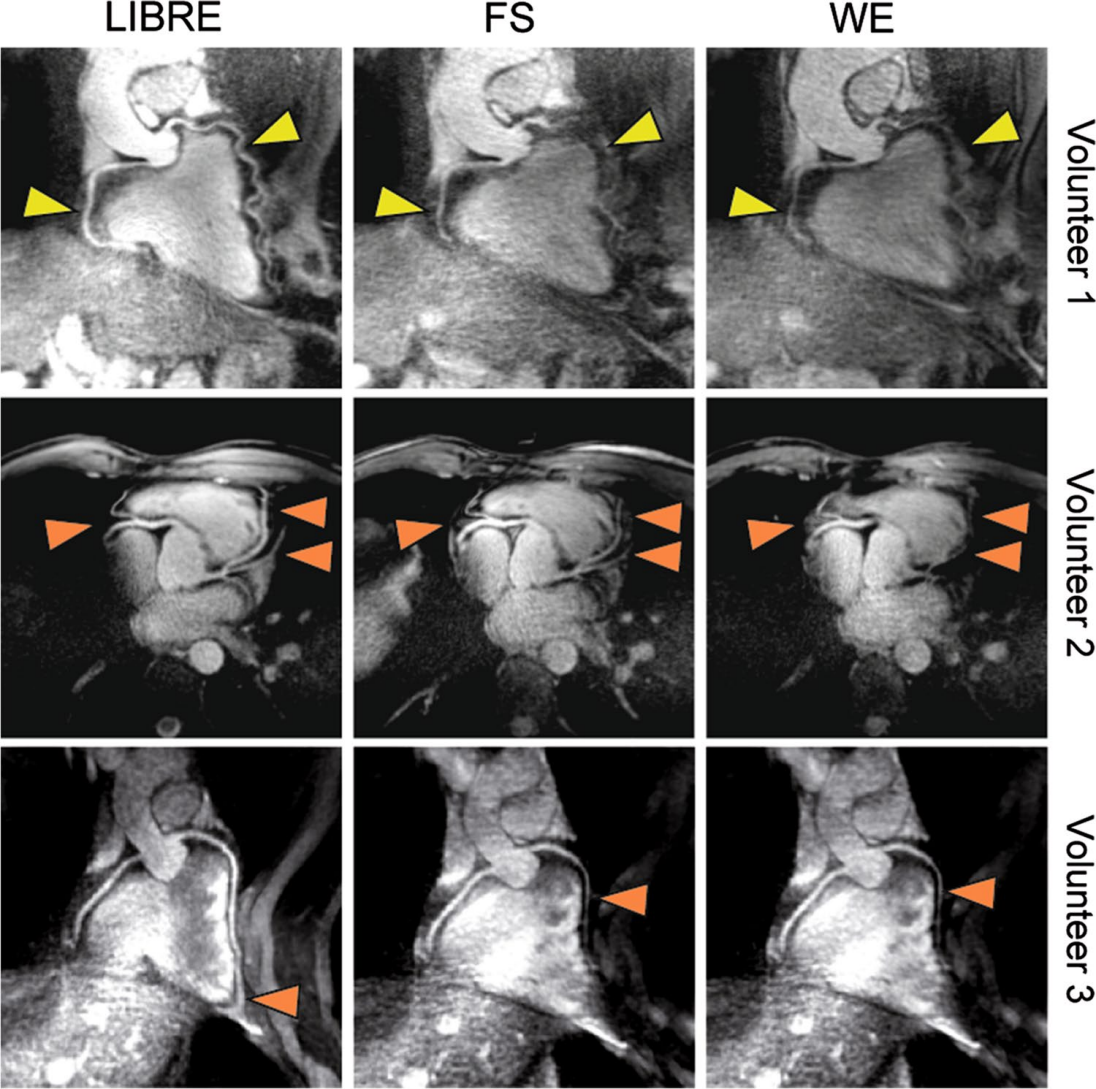
Comparison of the different fat saturation methods on radial trajectories coronary MRA at 3 T in healthy subjects. Coronary MRA images show the left and right coronary artery system depicting the RCA and the LAD in several subjects. Using the LIBRE pulse the visualization of the RCA and LAD was improved (yellow arrow), as well as fat suppression (orange arrows) compared with FS and WE. Vessel sharpness as well as imaged vessel length was significantly increased using LIBRE. Window and level are identical in images acquired in each volunteer. *MRA* magnetic resonance artery, *RCA* right coronary artery, *LAD* left anterior descending artery, *LIBRE* lipid insensitive binomial off-resonant excitation, *FS* fat saturation, *WE* water excitation. (Reprinted with permission from Batiaansen et al. [59] Copyright © 2019 by the Authors)

Non-contrast coronary MRA leverages the natural T2 differences between the blood and the surrounding architectures. Techniques such as fat saturation pre-pulses, magnetization pre-pulses, and T2 preparatory pulses augment the relative signal of the coronary arteries. These pre-pulses differentiate oxygenated blood in coronary arteries from the surrounding short T2 relaxation tissues such as cardiac muscle, deoxygenated blood in cardiac veins, and epicardial fat [55, 56]. Dixon water–fat separation [57] and lipid insensitive binomial off-resonant excitation (LIBRE) [58, 59] (Fig. 4) are also available options for fat suppression on coronary MRA. Another approach to fat suppression, the fast interrupted steady-state (FISS) sequence, uses an RF excitation pulse to natively suppress the fat signal without the need for periodical application of fat suppression and ramp-up pulses, and is reported to present a strong suppression of pericardial fat signal [60, 61]. The fat suppression technique is more challenging in radial imaging with higher magnetic field strengths, since the field inhomogeneities are typically accentuated. At the current stage, there is no conclusion on which fat suppression technique is the best for the current coronary MRA imaging technique. In comparison to the SSFP sequence acquisition in 1.5 T MRI, the T1 differences between blood and myocardium are smaller in 3 T MRI with gradient echo sequence acquisition. Therefore, contrast-enhanced coronary MRA was preferred during the period that 3 T coronary MRA acquisition was under development. With the maturation of the technique, the non-contrast coronary MRA image is more feasible and preferred in 3 T MRI.

The diagnostic performance of coronary MRA varies between studies, likely a result of the presence or absence of contrast administration, heterogeneity of the acquisition sequences, and the analytic methods used. A meta-analysis of 1638 patients in 24 studies including 1.5 T and 3 T coronary MRA studies reported the estimated sensitivity and specificity for detecting > 50% stenosis is 95% and 77% for contrast coronary MRA, while those values were 87% and 69%, respectively, for non-contrast coronary MRA [3]. The diagnostic performance of 1.5 T non-contrast coronary MRA to detect > 50% stenosis, with CAG serving as the reference standard, was reported by Kato et al. in a multicenter trial of 137 patients across seven hospitals in Japan. On a per-patient level, the observed sensitivity and specificity were 88% and 72%, respectively [12]. Hamdan et al. compared the performance of 3 T non-contrast coronary MRA against 64-slice coronary CTA to detect significant CAD, using quantitative coronary angiography as the gold standard. On a per-patient basis, the observed sensitivities and specificities were 87% and 77% for non-contrast coronary MRA versus 90% and 83% for CTA, respectively [10]. Despite the trend toward higher diagnostic accuracy values for CTA, both techniques were equal in their ability to identify patients who subsequently underwent revascularization. Current diagnostic performance of non-contrast coronary MRA is encouraging, but not as high as contrast coronary MRA and coronary CTA. However, as discussed earlier, the various advantages of non-contrast coronary MRA make it exceedingly attractive in many clinical situations. Technical developments provide further promise to improve the diagnostic performance of non-contrast coronary MRA.

## Current in-progress topics in coronary MRA

The major challenges of current coronary MRA sequences remain in: (1) unpredictable and long scan times mainly due to low gating efficiency which need image acquisition acceleration techniques including trajectory design, sparse sampling, and reconstruction; (2) residual respiratory motion artifacts due to simplified motion models and translational motion correction only. Advanced non-rigid motion correction techniques can address not only the motion artifacts but the low gating efficiency problem by allowing for 100% scan efficiency; and (3) overall image quality problems including those that may be solved by resolution improvement and denoising. Table 1 is a summary of the current technical challenges facing wider adoption of coronary MRA and corresponding solutions offered by current techniques and promising techniques under development which are discussed in the following paragraphs.

**Table 1:** Summary of technical challenges on coronary MRA and corresponding solutions with current and promising techniques

Problems	Current techniques		Promising techniques	
	Methods	Characteristics	Methods	Characteristics
1. Time-consuming image acquisition	Parallel imaging (PI) [80–84]	Well-established method Limitation of the acceleration factor Characteristic artifacts	Compressed Sensing (CS) [15, 16, 85, 86]	Potentially more effective in higher-dimensional image (3D > 2D) which is ideal for 3D coronary MRA acquisition Further techniques that combine PI and CS [65, 78, 88–91] or sparse k-space acquisition and DL [92, 93] are potentially available
2. Low scan efficiency from respiratory and ECG gating	Respiratory and ECG gating are the current standard technique	Strict gating gives better image quality but trade-off with long acquisition time	Self-gating with motion correction [17, 46, 75, 94–98] Golden-angle image acquisition and reconstruction [18, 71, 72, 91]	No need for respiratory gating or ECG gating Simultaneously acquired CINE images Time consuming for the reconstruction
3. Low resolution	Sub-millimeter spatial resolution acquisition [84]	Trade-off with SNR	Super-resolution with inter-slice reconstruction [99] Super-resolution based on overcomplete dictionaries [100, 104] Super-resolution based on deep learning [101, 102]	Inter-slice reconstruction method works well with 2D images
4. Noise	Mathematical denoising [108, 109]	No generally accepted standard for clinical use for any commercial filter	DL denoising [19, 20, 113–116, 140]	Better preservation of the edge Potentially works well with specific situation such as coronary MRA-dedicated denoising

*MRA* magnetic resonance angiography, *SNR* signal-to-noise ratio, *DL* deep learning, *PI* parallel imaging, *CS* compressed sensing, *ECG* electrocardiography

### Image acquisition acceleration technique (1): trajectory design and sparse sampling

Trajectory design of k-space sampling method is the first step of the image acquisition acceleration strategy and it is closely related with the subsequent reconstruction strategy. Cartesian k-space sampling is the most widely used trajectory design in current MRI image and in coronary MRA as well. The conversion from k-space domain to the image domain is simple with the inverse fast Fourier transform (FFT). Meanwhile, other types of non-Cartesian trajectory designs like radial and spiral trajectories have several advantages in the aspect of accelerated image acquisition and are of great interest. Reconstruction from non-Cartesian trajectories generally use filtered back-projection [62] or interpolation to the Cartesian grid k-space [17, 18, 63–65] so that the conversion to the image domain is more complicated.

Radial and spiral-like Cartesian trajectories have undersampling properties to create incoherent noise-like artifacts, which are a requirement for compressed sensing (CS) or low-rank reconstructions while still allowing for much shorter reconstruction times compared to non-Cartesian trajectories [65–69]. These techniques have been extensively used in recent coronary MRA studies.

Non-Cartesian trajectories can sample denser in the center of the k-space referring their design. This is preferable to image coronary MRA (1) to achieve faster acquisition with sparse k-space sampling and (2) to address motion artifact by average effect [70–72] or (3) to extract motion signals for self-navigation or motion compensation [46, 73–75]. “Golden-angle” radial sampling [76] is a good example of non-Cartesian trajectory. This design brings approximately uniform k-space coverage for many useful subsets of acquired data, which enables dynamic imaging studies with continuous data acquisition and retrospective reconstruction of image series with flexible temporal resolution by grouping a different number of consecutive measurements into each temporal frame [77, 78]. Furthermore, the “stack-of-stars” k-space sampling is the hybrid of golden-angle radial trajectories with Cartesian sampling. By combined application of parallel imaging to the Cartesian sampling direction and compressed sensing to the remaining directions for the reconstruction, streaking artifacts can be mostly removed with improved delineation of fine structures using the proposed strategy [71, 78, 79]. In the following discussion on reconstruction and motion correction techniques, the relevant trajectory designs are discussed together.

### Image acquisition acceleration technique (2): reconstruction (parallel imaging and compressed sensing)

Sparse k-space sampling which violates the Nyquist sampling theorem is central to accelerated image acquisition. Two major reconstruction methods are parallel imaging (PI) and CS, which are in practice combined to achieve highly accelerated acquisition.

Parallel imaging (PI) is currently the most widely used method for image acquisition acceleration. PI approaches share the following characteristics: (1) undersampled k-space data in the phase-encoding direction (and partition-encoding direction in 3D imaging), (2) data acquisition with an array of independent receiver channels instead of using a large homogenous volume receive coil, and (3) usage of a dedicated algorithm, which requires some knowledge of the individual coil sensitivities, to combine the undersampled data [80–83]. Gharib et al. have reported that PI combined with high-resolution coronary MRA results in shortened image acquisition times with preserved image quality [84]. Drawbacks of PI include the limitation of the maximum acceleration factor caused by the number of receiver channels and specific artifacts, such as residual aliasing and g-factor noise enhancement [83].

CS is a promising image acquisition acceleration method that works more efficiently in higher dimensional images such as 3D images or images with a temporal dimension. The key components of CS are: (1) image sparsity or transform sparsity, (2) pseudo-random undersampling, and (3) iterative nonlinear reconstruction [85]. CS reconstructions require prior optimization of a regularization parameter, or data consistency tuning constant, to find the best trade-off between the data consistency and sparsity terms [85]. Sparse and random sampling in multi-dimensional data sets result in ‘noise-like’ incoherence artifacts unlike coherence artifacts seen in PI without random sampling [86]. These incoherence artifacts can be reduced within the CS reconstruction, providing an ideal condition for 3D coronary MRA image acquisition. Coronary MRA acquired with CS has shown comparable image quality with PI-coronary MRA, yet with shortened image acquisition time [15]. Akçakaya et al. have compared conventional PI and CS combined with LOST de-aliasing strategy [87] for sub-millimeter whole-heart coronary MRA. Overall image quality and perceived (semi-quantitative) SNR of the CS images were significantly higher than those of conventional PI [16].

Further image acceleration methods which combine CS with PI such as the k-t sparse technique with sensitivity encoding (SENSE) reconstruction [88], iterative Golden-angle RAdial Sparse Parallel MRI (iGRASP) [78], self-consistent parallel imaging reconstruction (SPIRiT) [65] and parallel imaging using eigenvector maps (ESPIRiT) [89] are areas of active research. SPIRiT and ESPIRiT are able to incorporate an L1-norm minimization term to additionally enforce sparsity in a transform domain, which has the same underlying theory as CS [90]. Haris et al. have reported high quality of images with iGRASP in comparison to the PI-based real-time imaging in the cardiac and extra-cardiac structure visibility in fetal cardiac examinations [91].

### Image acquisition acceleration technique (3): reconstruction (deep learning (DL))

**Fig. 5:**
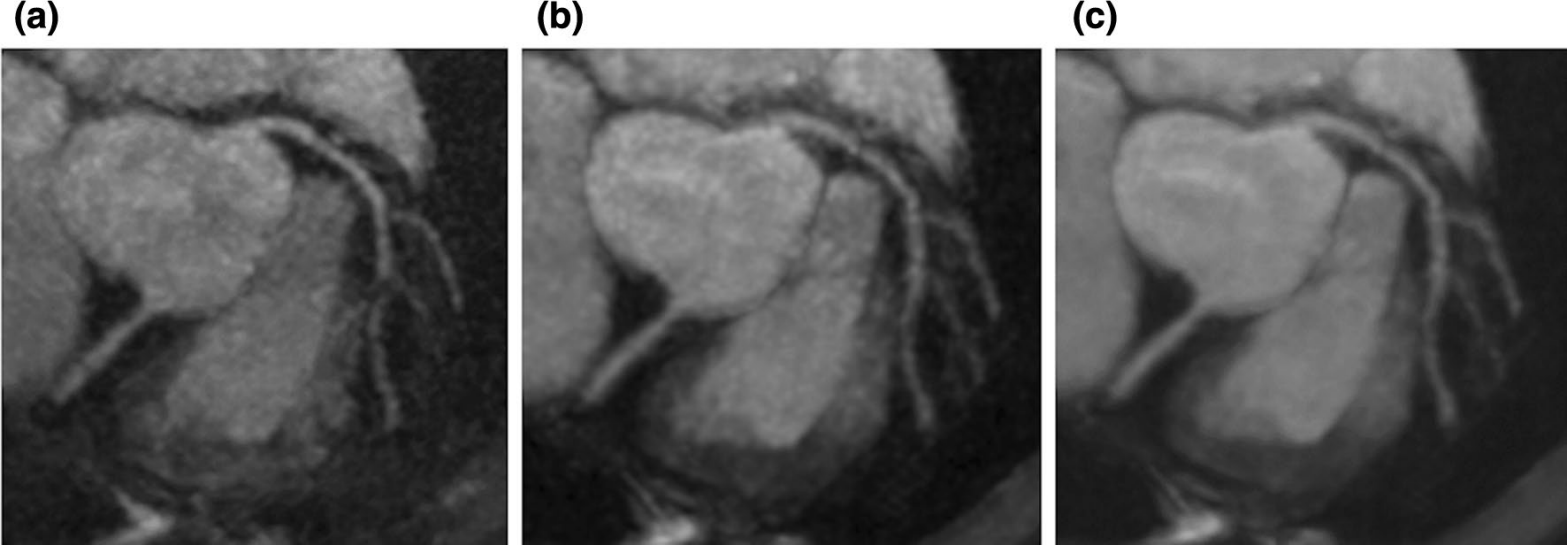
Coronary MRA images of PI, CS, and CS with deep learning reconstruction. Three MPR images with identical resolution yet different image acceleration methods and post-processing are shown. **a** PI. **b** CS. **c** CS with deep learning reconstruction postprocessing. **c** The best image quality among the three images. The PI and CS images were acquired with a spoiled gradient echo sequence with ECG-gating, diaphragm navigator gating, and fat suppression with spectral attenuated inversion recovery. The MRI acquisition parameters were FOV = 380 × 380 mm, Matrix = 392 × 384, slice thickness = 1.0 mm, slice number = 152, acceleration factor = 2.0 × 2.0, TR = 5.3 ms, TE = 2.0 ms, flip angle = 12 degree, bandwidth = 279 Hz/pix, acquisition voxel size = 1.0 × 1.0 × 1.0 mm, reconstructed voxel size = 0.5 × 0.5 × 0.5 mm, navigator gating window = 4 mm. For the DLR technique, see Refs. [116, 140]. *MRA* magnetic resonance angiography, *PI* parallel imaging, *CS* compressed sensing, *MPR* multiplanar reconstruction

Another approach is deep learning reconstruction from the subsampled k-space data. In a recent CS combined with DL approach, successful reconstruction was achieved from 29% of the k-space data with comparable image quality to fully sampled MRI reconstructions [92]. Combining tiny golden-angle radial sampling (tGA) with DL resulted in more than five times faster overall reconstruction time, superior image quality, and better accuracy of biventricular volumes than iGRASP when compared in short axis cine image. The total reconstruction time for all the short axis cine slices was 22.0 s with this method, which allowed for the real-time image reconstruction [93]. These advanced techniques all hold great potential for use in coronary MRA image acquisition acceleration and reconstruction, although there is no current consensus on which technique is most favorable. The central thesis is that the combination of PI and CS is expected to afford high spatial resolution while reducing the total time of acquisition. Additionally, deep learning reconstruction (DLR) promises effective denoising to improve SNR and CNR. Figure 5 shows a comparison between PI, CS, and CS processed with DLR.

### Motion correction

ECG gating and respiratory gating is the most widely used motion correction method. Its drawbacks are the low efficiency of data acquisition resulting in unpredictable and long scan times despite the need for expert planning. This respiratory gating method prospectively corrects for translational motion of the heart in the superior–inferior direction, while it does not account for remaining directions, or rotations or nonrigid deformations.

Several promising techniques are being investigated that aim to improve the motion artifact as well as the scan efficiency. One promising approach is a self-navigator derived from the imaging data itself [73]. Such self-gating methods including 2D and 3D image navigators have been first introduced for 3D single heart phase coronary MRA [94, 95].

**Fig. 6:**
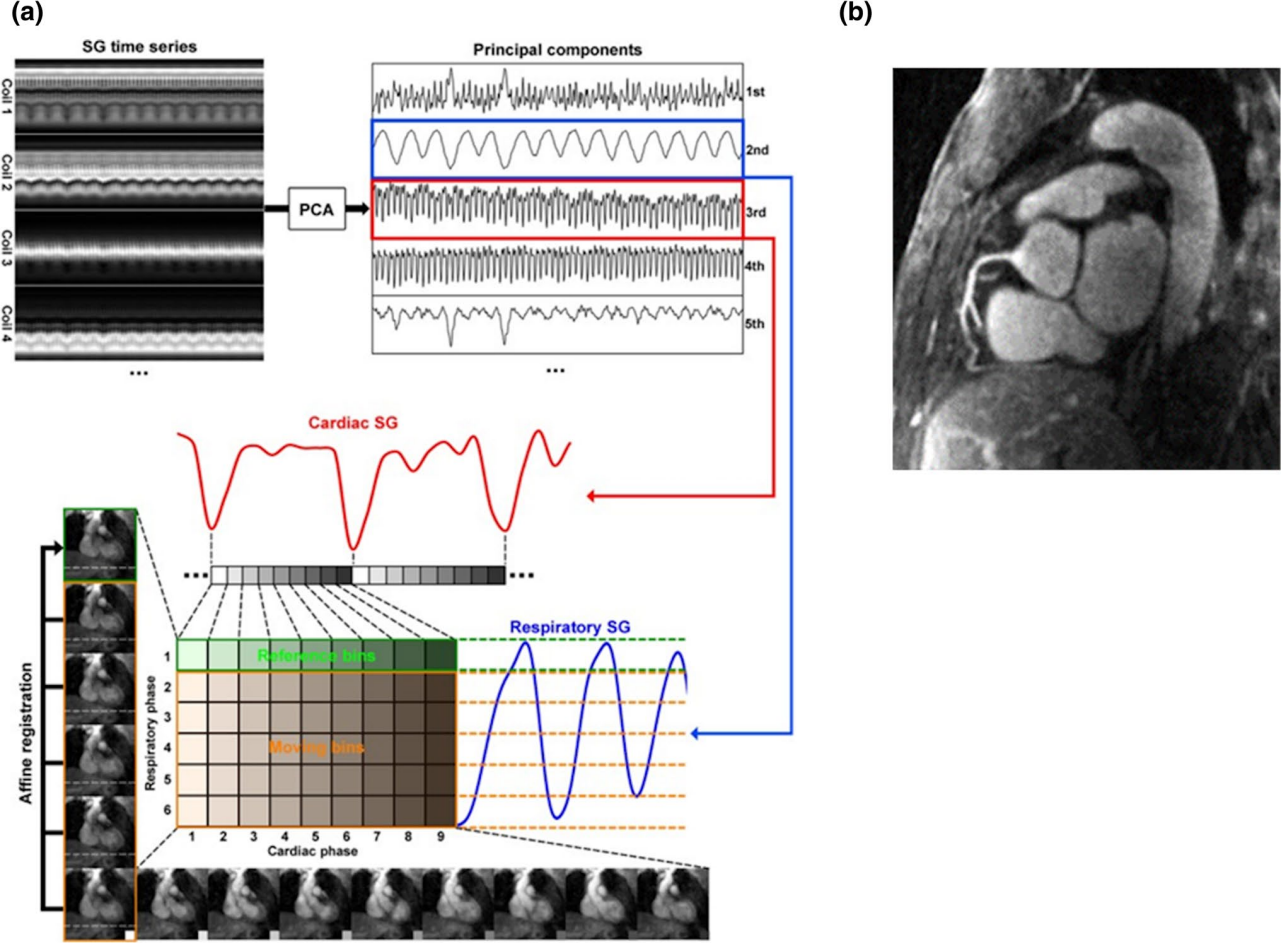
Schematics of the proposed self-gating, data binning, and respiratory motion correction framework. **a** First, the cardiac and respiratory motion components are identified from the PCA of the multichannel self-gating profile time series. Then, the imaging data are mapped to different cardiac and respiratory bins based on its cardiac and respiratory phase derived from the motion signals. Next, with one common respiratory phase selected as reference (in this example, respiratory phase 1 for cardiac phases 1–9), all other bins (respiratory phases 2–6, cardiac phases 1–9) are registered to the corresponding reference bin of the same cardiac phase using an affine transform model. The k-space trajectory and data are then modified accordingly for respiratory motion correction. The six images on the left show the six respiratory phases in cardiac phase 1. The horizontal dashed lines help visualize the SI motion of the heart due to respiration. The nine images on the bottom show the nine cardiac phases in respiratory phase 6. The contraction of the left ventricle can be clearly seen. **b** A representative case with excellent depiction of the right coronary artery. *PCA* principal component analysis. Reprinted with permission from Pang et al. [17] Copyright © 2014 by the Wiley Periodicals, Inc.

When combined with 3D affine or 3D non-rigid reconstruction, this approach can achieve 100% scan efficiency [17, 46, 96–98] (Fig. 6). Bhat et al. have investigated a whole-heart coronary MRA acquisition method with 100% scan efficiency reconstructed with respiratory motion correction. They used the navigator signal as a reference respiratory signal to segment the data into six respiratory bins. The reconstruction of low-resolution undersampled images for each respiratory bin was enabled from the 3D projection reconstruction k-space acquisition, which samples data on a spiral path running on the surface of a sphere. The data from different respiratory bins were retrospectively combined after motion correction based on the affine transform. When compared with a traditional navigator gating approach, their method reduced scan time by a factor of 2.5 while image quality was preserved [46]. Piccini et al. have reported that respiratory self-navigation with 100% acceptance rate significantly reduced the acquisition time from 16.23 ± 6.28 to 6.07 ± 0.57 min (*p* < 0.01) when compared with the navigator-gated coronary MRA acquisition [96]. In addition, the authors reported that the end-expiratory reference position significantly improved the image quality as compared to using end inspiration as a reference [97].

Another promising approach toward motion correction is the continuous 3D golden-angle radial sampling and reconstruction of separated cardiac and respiratory dimensions [18, 71, 72]. These methods have the advantage that coronary MRA images can be reconstructed with different temporal resolutions, or, since the images are acquired over the entire cardiac cycle, cine images of other structures can be derived from the same image data [17]. Another advantage is time reduction through elimination of the respiratory gating preparation. Feng et al. used a framework called eXtra-Dimensional GRASP (XD-GRASP) [71] which combines a continuous 3D golden-angle radial sampling scheme with a multidimensional compressed sensing technique to reconstruct separated cardiac and respiratory dimensions. The proposed method resulted in higher image quality in the myocardium and coronary arteries, better coronary sharpness, and longer coronary length visualized than respiratory motion-corrected 3D and 4D whole-heart imaging [18]. Haji-Valizadeh et al. have scanned the aorta with an accelerated coronary MRA sequence with stack-of-stars k-space sampling and GRASP reconstruction, achieving comparable image quality as contrast-enhanced conventional imaging with significant scan time reduction (5:55 ± 0:48 min vs. 6:56 ± 2:10 min). The mean off-line image reconstruction time was 4 h 41 min and 13 s [72].

While these methods look promising, their robustness in daily practice with respect to reconstruction with clinically acceptable reconstruction time and motion correction is an ongoing area of research [17].

### Image quality improvement (1): high-resolution coronary MRA acquisition/super-resolution coronary MRA post-processing

Coronary MRA image acquisition under free breathing offers the opportunity for improved spatial resolution including sub-millimeter coronary MRA, while there are drawbacks such as increased acquisition times and lower SNR. Gharib et al. acquired high-resolution coronary MRA using 3 T magnetic resonance imaging (MRI) with a voxel size as small as 0.35 × 0.35 × 1.5 mm^3^ and compared the images with coronary MRA images having 0.7 × 1 × 3mm^3^ voxel size. The higher resolution images showed a 47% improvement in vessel sharpness, although image acquisition times were longer and SNR and CNR reduced as compared to the lower-resolution coronary MRA [84]. Super-resolution (SR) is a different approach than voxel size adjustment and has been already utilized in brain imaging. There are several methods reported such as inter-slice reconstruction applied to 2D multislice MRI [99], image domain SR via patch-based sparse representation using overcomplete dictionaries [100], and deep learning-based SR [101–103]. In coronary MRA images, dictionary-based super-resolution applied to 1.5 T non-contrast coronary MRA was reported by Ishida et al. [104]. Their SR technique showed significant improvement in the detection of coronary artery stenosis as compared to conventional resolution coronary MRA. Further improvements in acquisition and reconstruction methods are likely to lead to routine high-resolution coronary MRA images.

### Image quality improvement (2): denoising/smoothing

Coronary MRA image quality is not merely assessed using SNR or image resolution measures. Other metrics are also considered, such as overall visual image quality allowing meaningful clinical diagnosis, the visually recognizable coronary length, and the crispness/sharpness (conspicuity) of the coronary wall edges [15]. Such a comprehensive image quality assessment has traditionally relied on experienced observers, but initial results from deep learning architecture for automated image quality assessment are promising. Efforts are already ongoing in other domains such as liver MRI [105] and in other modalities such as coronary CTA [106], and these developments may be applicable to coronary MRA as well.

Image denoising is an indispensable first step in many practical applications including coronary MRA. It aims to preserve edges while smoothing out the noise. Oversmoothing renders the image blurry, which is problematic in coronary MRA for the interpretation of clinically meaningful findings such as intensity change in the coronary lumen or fine anatomies like distal coronary arteries or branches. Noise may be roughly categorized into additive white Gaussian noise, multiplicable noise (speckle noise), impulse noise (salt and pepper noise), and shot noise (Poisson noise). In MR images, the predominant noise is actually non-Gaussian such as a Rician distribution [107], yet it is a common practice to assume noise as Gaussian since Rician noise asymptotically becomes Gaussian for high SNR [107]. Some denoising techniques aim to cover a wide range of noise models such as Gaussian and non-Gaussian noise [108, 109]. In the paragraphs below we discuss two denoising approaches, namely (1) conventional methods and (2) deep learning approaches applicable for coronary MRA imaging.

#### Conventional methods

There is no generally accepted standard for clinical/commercial application of any denoising filter. Denoising filters applied to coronary MRA images need to be carefully considered, with regard to the balance between preserving the detailed coronary information and strength of denoising. Most traditional spatial filtering techniques directly operate on pixels in the image (or spatial) domain and have a tendency to blur the edges, which is not preferred in coronary imaging. In contrast, transform domain filtering operates on the wavelet transformed data and then transforms it back to the spatial domain, leading to faster computation and preserved edge-detail fidelity based on at least one prior report [110]. Most filtering techniques assume an equal noise distribution across the image, although spatially varying noise levels occur such as those obtained by parallel imaging. In such cases, spatially adaptive non-local denoising may provide better results [108]. One example of such denoising methods and the current state of the-art is block matching and 3-D filtering (BM3D) [109]. That method groups similar patches into blocks, transforms them to wavelet coefficients, then thresholds to obtain an optimal representation, and transforms back. BM3D can be adapted to various noise models such as additive noise and non-Gaussian noise.

#### Deep learning approaches

**Fig. 7:**
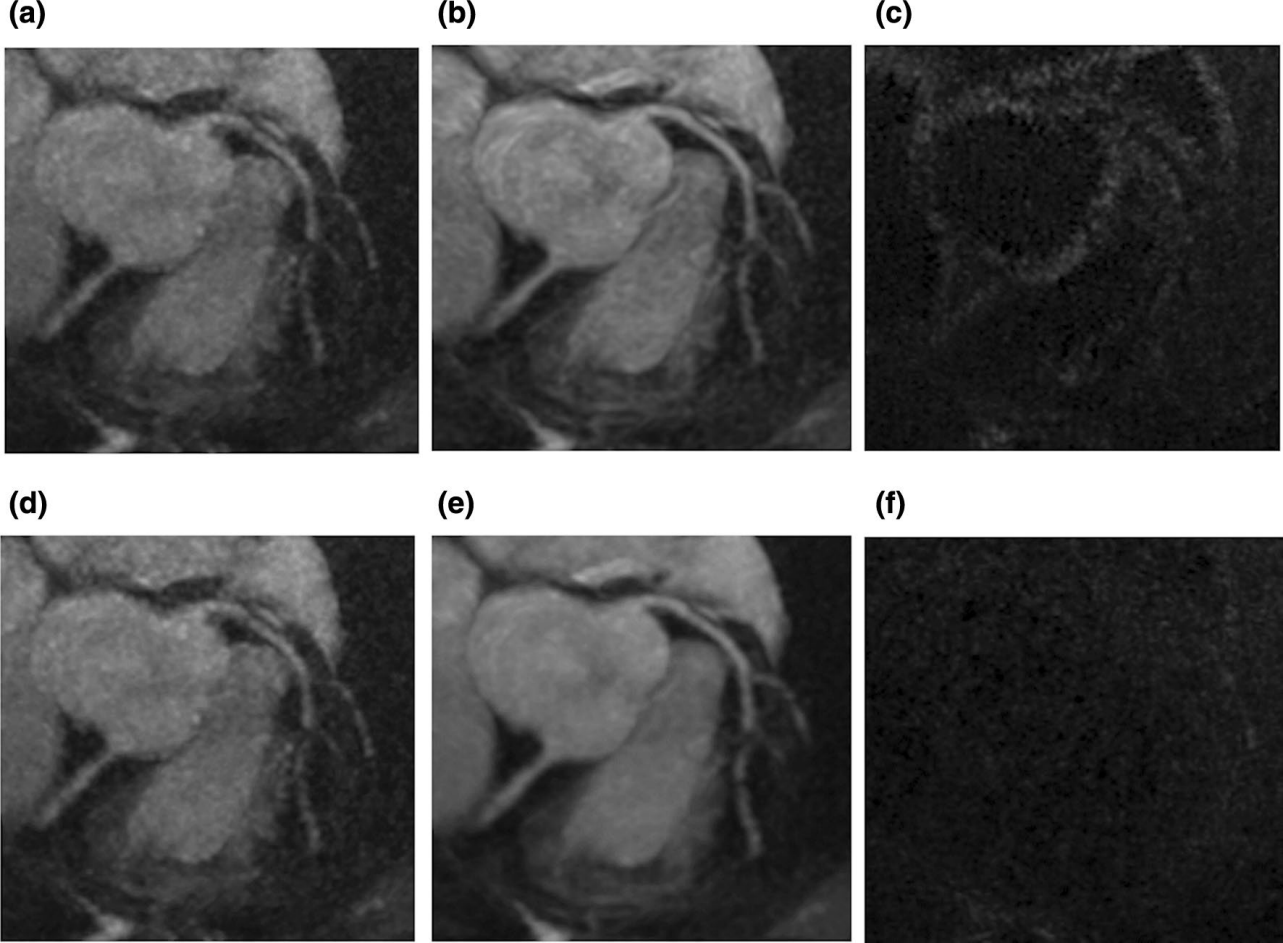
Comparison of subtracted noises by conventional denoising and DLR on the same coronary MRA data set. **a**, **d** Same original coronary MRA images acquired with parallel imaging acceleration technique. **b** Coronary MRA image after conventional denoising. **e** Coronary MRA image after DLR. **c** The subtracted noise between **a** and **b**. **f** The subtracted noise between **d** and **e**. All the panels **a**–**f** are in the same WL and WW. Subtraction images show that conventional denoising removes the edges from the original coronary MRA image (**c**), while DLR works on noise and retains the edge information (**f**). Consequently, the signal-to-noise ratio is higher in DLR processed image (**e**) than the conventional denoised image (**b**). The original image was acquired with a spoiled gradient echo sequence with ECG-gating, diaphragm navigator gating, and fat suppression with spectral attenuated inversion recovery. The MRI acquisition parameters were FOV = 380 × 380 mm, matrix = 392 × 384, slice thickness = 1.0 mm, slice number = 152, acceleration factor = 2.0 × 2.0, TR = 5.3 ms, TE = 2.0 ms, flip angle = 12 degree, bandwidth = 279 Hz/pix, acquisition voxel size = 1.0 × 1.0 × 1.0 mm, reconstructed voxel size = 0.5 × 0.5 × 0.5 mm, navigator gating window = 4 mm. For the conventional filtering, GA01 filter was used. For the DLR technique, see references [116, 140]. *DLR* deep learning reconstruction, *MRA* magnetic resonance angiography, *WL* window level, *WW* window width, *GA* gain algorithm

Deep learning reconstruction does not require modeling of noise. It has the ability to perform improved denoising through efficient optimization of the denoising level and good edge preservation based on the characteristics of “learning noise” strategy, which works well on coronary MRA images (Fig. 7). Consequently, denoising with deep learning may achieve higher SNR values than conventional filters (Fig. 7). Another characteristic is its flexibility with various frameworks, including application to the image domain as well as the k-space domain to create either denoised spatial images or denoised k-space data [111, 112]. Learning a specific type of noise in advance such as in a trainable nonlinear reaction diffusion (TNRD) model [113] would restrict its performance to the specified forms of prior learning and accordingly limited in blind image denoising. To overcome this problem, feedforward denoising convolutional neural networks (DnCNNs) were proposed by Zhang et al., which are capable of handling Gaussian denoising with unknown noise levels [114]. Isogawa et al. proposed an adaptive approach by using soft shrinkage for the activation function of deep CNN resulting in a noise adaptive algorithm [115]. Furthermore, in one implementation [116], the application of 7 × 7 discrete cosine transform (DCT) convolution to extract higher frequency components followed by deep learning-based CNN using soft shrinkage for adaptive denoising successfully removed noise regardless of SNR parameters such as contrast settings, matrix size, 2D and 3D, among other variables.

While still in its infancy, the use of deep learning strategies shows promise in denoising coronary MRA images, where fine anatomical images require good edge preservation and high SNR [19, 20]. Its flexibility with various noise levels and noise types is a great advantage when scanning patients with different body sizes. Its potential application to different frameworks may be valuable when combined with image acquisition acceleration techniques. However, care must be taken with regard to the application of deep learning reconstruction methods, since inappropriate denoising may cause artificial image manipulation leading to a loss of accuracy in image interpretation and reported clinical findings. Further validation is therefore necessary before adoption.

## Opportunities for utilization from research-based modality to clinically utilized modality

At present, there is no standardization of coronary MRA assessment methods. Currently available research-based methods are summarized in Table 2, and will be discussed in the following.

**Table 2:** Summary of research-based coronary MRA assessment methods

Target of the assessment		Methods
1. Stenosis severity		Signal intensity drop quantification [119, 120]
2. Plaque characteristics		MPRAGE [122, 123, 141] and T2* [124, 125] images for the iron accumulation detection in vulnerable plaques Ultrashort TE (UTE) for fibrosis and calcification detection [126, 127] Susceptibility weighted imaging for calcification detection [128]
3. Physiological function		Phase contrast-based coronary blood flow, coronary sinus flow, and coronary flow reserve measurement [130, 132–137] Pressure gradient along the stenotic lesion [138] 4D flow [139]

*MRA* magnetic resonance angiography, *MPRAGE* magnetization-prepared rapid acquisition with gradient echo, *UTE* ultrashort echo time

### Software development for the assessment of coronary MRA images

While there are many multipurpose cardiovascular analysis packages, there are few dedicated non-contrast coronary MRA assessment tools commercially available or widely used. Coronary tree tracking and stenosis assessment remain a labor-intensive and time-consuming process. There are several specific reasons for this: (1) low SNR of the coronary lumen, (2) vulnerability of the coronary MRA images to artifacts that hamper auto-tracking of the vessels, and (3) rather low resolution of the image that prevents tracking of the smaller side branches. Attempts such as the “soap-bubble method” which assumed the coronary tree distribution on a relatively smooth 3D surface of a soap bubble to derive the final 2D maximum intensity projection image [117] or coronary MRA vessel centerline tracking and boundary segmentation based on geometric deformable models and optimized energy forces [118] have been previously reported. However, the application of these techniques in different clinical settings as well as their robustness remains untested.

### Coronary MRA stenosis assessment by signal intensity (SI) drop quantification

**Fig. 8:**
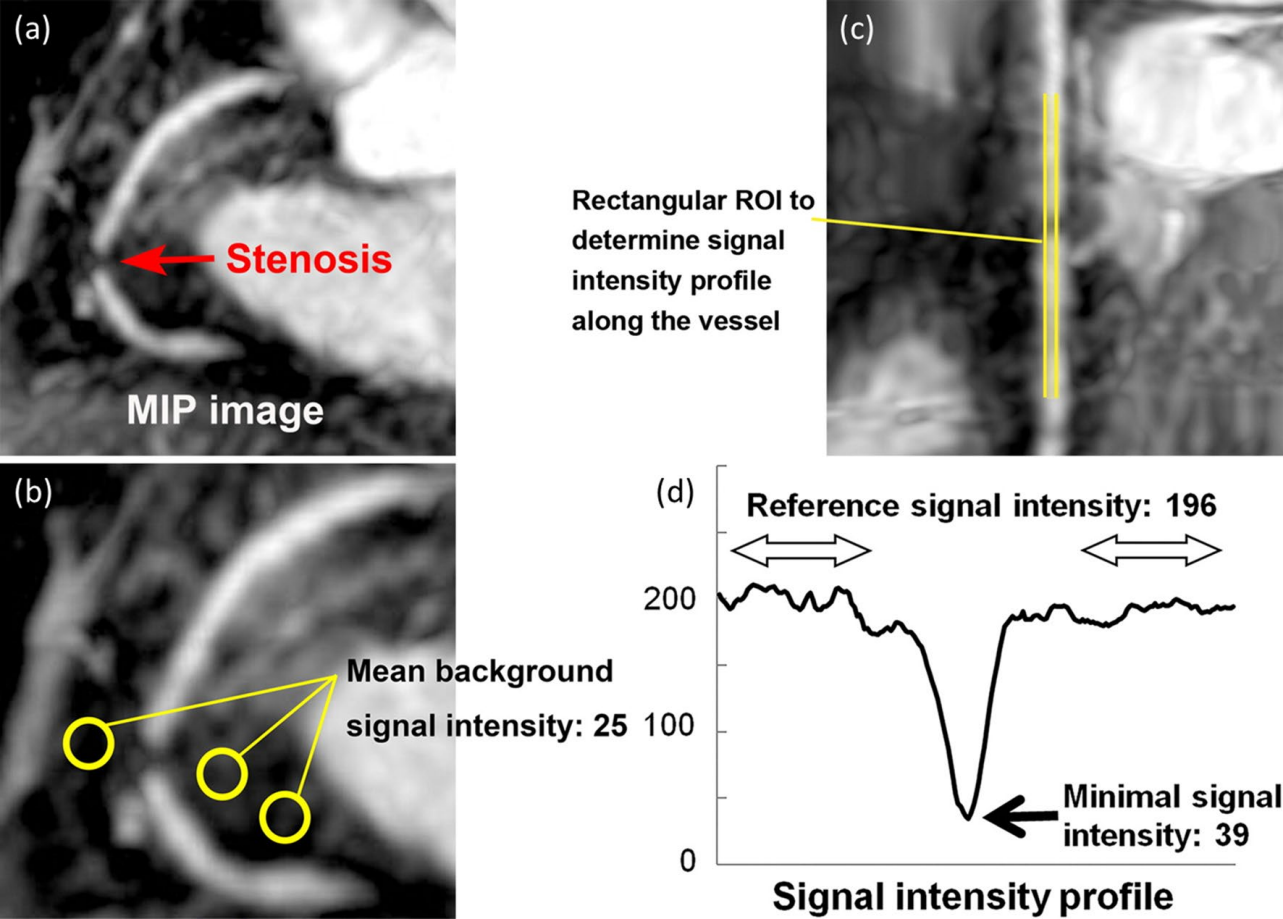
QA of narrowing in the coronary artery on the basis of the signal intensity profile along the vessel. **a** MIP image of the major coronary vessel was generated from a three-dimensional coronary MRA. **b** Background signal intensity was measured by placing three ROIs (yellow circles) in pericardial fat on the image and averaging signal intensity values in three ROIs. **c** A stretched multiplanar reconstruction image was reconstructed along the artery, and signal intensity was determined by placing an elongated rectangular ROI with a width of 3 pixels along the vessel lumen (yellow elongated rectangular ROI). A luminal signal-intensity profile was generated after subtracting the background signal intensity. **d** The percentage stenosis of the coronary artery was determined by measuring the minimal signal intensity at the stenotic lesion and the signal intensity at a reference location without stenosis. In this case, background signal intensity was 25, reference signal intensity was 196 (221–25), minimal signal intensity was 39 (64–25), and percentage stenosis with QA of coronary MRA was 80.1% [1 − (39/196) × 100]. *QA* quantitative analysis, *MIP* maximum intensity projection, *MRA* magnetic resonance angiography, *ROI* region of interest. (Reprinted with permission from Yonezawa et al. [119] Copyright © 2013 by RSNA)

Coronary MRA interpretation is usually performed visually, but its quantification is important for the generalization of the method. A previous study investigated the signal intensity (SI) profile across the coronary artery and reported an SI drop of 35% corresponding to significant stenosis by CAG (Fig. 8) [119]. Notably, this SI drop was not observed in chronic total obstruction cases [120]. One possible explanation is that the SI is affected not only by the stenosis severity, but also by the plaque characteristics.

### Plaque assessment

**Fig. 9:**
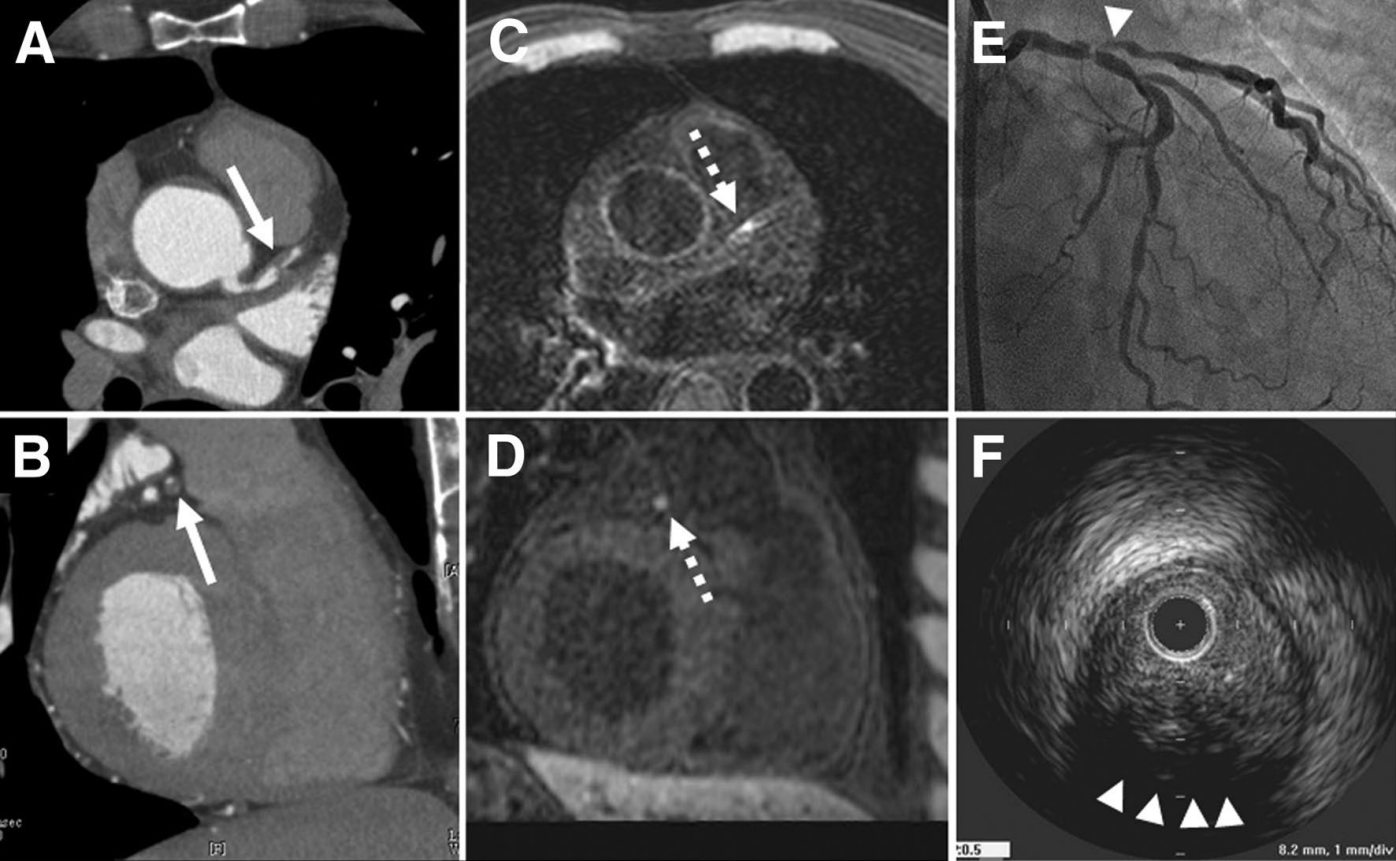
Representative case of HIP in the proximal LAD is presented. Coronary CTA (**a** horizontal, **b** sagittal) demonstrates the low-density positive remodeling plaque (− 32 Hounsfield units, remodeling index: 1.27) (arrow) with severe coronary stenosis in the proximal LAD. On the corresponding CMR (**c** horizontal, **d** sagittal), this low-density plaque was visualized as a “hyperintense spot” (dashed arrow). On the coronary angiography, severe coronary stenosis was observed (**e**) (arrowhead), and on IVUS examination (**f**), positive remodeling plaque (remodeling index: 1.29) with ultrasound attenuation (arrowheads) was observed in the proximal LAD, corresponding to the plaque observed by both coronary CTA and CMR. *HIP* hyperintense plaque, *LAD* left anterior descending artery, *CTA* computer tomography angiography, *CMR* cardiac magnetic resonance, *IVUS* intravascular ultrasound. (Reprinted with permission from Kawasaki et al. [121] Copyright © 2009 by the American College of Cardiology Foundation)

Various sequences have been investigated for plaque assessment, though none of these techniques are currently viable clinically. T1-weighted magnetization-prepared rapid acquisition with gradient echo (MPRAGE) (Fig. 9) [121–123] and T2* [124, 125] images describe iron accumulation in the vulnerable plaque. Noguchi et al. have reported that the presence of high-intensity plaque (HIP) on MPRAGE image was significantly associated with coronary events (*p* < 0.0001, HR = 3.15) [122]. HIP detection prior to PCI was reported to be clinically relevant to avoid no-reflow phenomenon [123]. Ultrashort echo time (UTE) [126, 127] and susceptibility weighted imaging [128] are used to improve the visualization of calcified plaque. The distribution of iron and calcium is under research interest, since iron is suspected to accelerate the progression of atherosclerotic lesions while suppressing its calcification, and alternatively calcification could defend against atherosclerotic progression by excluding iron [129].

### Physiological assessment of the coronary arteries

Physiological coronary function assessment by MRI uses phase-contrast (PC) imaging to quantify the blood flow through the coronary arteries. Sakuma et al. have reported in their canine study that the coronary blood flow (CBF) measured with the PC technique correlated well with flowmeter measurements [130]. Flow in the coronary sinus, which represents 96% of the total myocardial blood flow [131], has also been assessed [132, 133]. Lund et al. have reported in a canine model that CBF measured in the left anterior descending (LAD) and left circumflex (LCX) showed excellent correlation with the flow of coronary sinus (*r* = 0.98, *p* < 0.001) [132]. Coronary flow reserve (CFR) which is the fractional CBF increase induced by stress agent is another target. Sakuma et al. have reported that the CFR measured in LAD in healthy subjects was significantly higher than that in patients with significant LAD stenosis [134]. Kato et al. have reported that in patients with suspected coronary artery disease, CFR assessed on the coronary sinus flow showed higher hazard ratio (HR) for the prediction of major adverse cardiac events than the presence of > 10% ischemia on stress perfusion cardiac magnetic resonance (HR: 14.16 vs 6.50, respectively) [133]. These physiological measurements estimate the severity and extent of atherosclerotic burden, including the presence of multi-vessel disease or microvascular dysfunction [135, 136]. The prognostic value of CFR [133, 135–137] is noted and it can be a good candidate for a “non-contrast one-stop-shop-test” to be combined with coronary MRA.

Meanwhile, another measurement of coronary flow, the functional flow ratio (FFR), is still under development. Direct assessment of the pressure gradient (Δ*P*) along the stenotic coronary artery with PC imaging was reported by Deng et al., who observed a significant increase in Δ*P* in suspected coronary stenosis lesions than in controls (6.40 ± 4.43 mmHg vs. 0.70 ± 0.57 mmHg, *p* = 0.025) [138]. Current technical problems related to this PC application are the partial volume effects at stenotic regions, possible impact from turbulence on the accuracy of PC-MRI velocity measurements, and the need to cope with cardiac and respiratory motion [138]. 4D flow is an advanced promising technique of PC with flow encoding in all three dimensions of space, plus time along the cardiac cycle. The current typical spatial resolution of 4D flow is 1.5 × 1.5 × 1.5 to 3 × 3 × 3 mm^3^ [139], which is not enough for coronary artery imaging. Further developments with higher resolution are expected.

## Conclusions

Non-contrast coronary MRA is a non-invasive, non-ionizing radiation modality that is particularly unique, as it does not require contrast use to enhance intra-luminal blood flow. These characteristics have great potential for routine clinical applications. We reviewed the current clinical use and the in-progress technical developments that could significantly impact coronary MRA prospectively. The central thesis is that the combination of PI and CS is expected to maintain high spatial resolution while reducing the total time of acquisition, and application of DLR is promising for effective denoizing to improve SNR and CNR. Research-based coronary MRA assessment methods of stenosis severity, physiological function, and plaque characteristics are being developed to transform coronary MRA into a robust non-invasive imaging modality to be used routinely for clinical decision making in cardiovascular medicine.

## Abbreviations

MRA: Magnetic resonance angiography
CAD: Coronary artery disease
CTA: Computed tomography angiography
CAG: Coronary angiography
LGE: Late gadolinium enhancement
ECG: Electrocardiography
SSFP: Steady-state free precession
RCA: Right coronary artery
SNR: Signal-to-noise ratio
CNR: Contrast-to-noise ratio
bSSFP: Balanced steady-state free precession imaging
FLASH: Fast low angle shots
MRI: Magnetic resonance imaging
SR: Super-resolution
BM3D: Block matching and 3-D filtering
TNRD: Trainable nonlinear reaction diffusion
DnCNNs: Denoising convolutional neural networks
PI: Parallel imaging
CS: Compressed sensing
SENSE: Sensitivity encoding
iGRASP: Iterative golden-angle radial sparse parallel MRI
SPIRiT: Self-consistent parallel imaging reconstruction
ESPIRiT: Eigenvector maps self-consistent parallel imaging reconstruction
tGA: Tiny golden radial
DLR: Deep learning reconstruction
XD-GRASP: Extra-dimensional GRASP
PC: Phase contrast
CBF: Coronary blood flow
LAD: Left anterior descending
LCX: Left circumflex
CFR: Coronary flow reserve
MPRAGE: Magnetization-prepared rapid acquisition with gradient echo
HIP: High-intensity plaque
UTE: Ultrashort echo time
